# Application of Multivariable Analysis and FTIR-ATR Spectroscopy to the Prediction of Properties in Campeche Honey

**DOI:** 10.1155/2016/5427526

**Published:** 2016-12-14

**Authors:** Francisco Anguebes, Lucio Pat, Bassam Ali, Armando Guerrero, Atl V. Córdova, Mohamed Abatal, José P. Garduza

**Affiliations:** ^1^Facultad de Química, Universidad Autónoma del Carmen, Calle 56 No. 4 Esquina Avenida Concordia, Colonia Benito Juárez, 24180 Ciudad del Carmen, CAM, Mexico; ^2^Colegio de la Frontera Sur, Av. Rancho Polígono 2-A, Ciudad Industrial, 24500 Lerma, CAM, Mexico; ^3^Facultad de Ingeniería, Universidad Autónoma de Yucatán, Av. Industrias no Contaminantes por Periférico Norte, Cordemex, 150 Mérida, YUC, Mexico; ^4^Colegio de Postgraduados, Campus Tabasco, Periférico Carlos A. Molina s/n, 86500 Cárdenas, TAB, Mexico; ^5^Facultad de Ingeniería, Universidad Autónoma del Carmen, Campus III, Avenida Central S/N, Esq. con Fracc. Mundo Maya, 24115 Ciudad del Carmen, CAM, Mexico

## Abstract

Attenuated total reflectance-Fourier transform infrared spectrometry and chemometrics model was used for determination of physicochemical properties (pH, redox potential, free acidity, electrical conductivity, moisture, total soluble solids (TSS), ash, and HMF) in honey samples. The reference values of 189 honey samples of different botanical origin were determined using* Association Official Analytical Chemists, (AOAC),* 1990;* Codex Alimentarius*, 2001*, International Honey Commission,* 2002, methods. Multivariate calibration models were built using partial least squares (PLS) for the measurands studied. The developed models were validated using cross-validation and external validation; several statistical parameters were obtained to determine the robustness of the calibration models: (PCs) optimum number of components principal, (SECV) standard error of cross-validation, (*R* ^2^_cal_) coefficient of determination of cross-validation, (SEP) standard error of validation, and (*R* ^2^_val_) coefficient of determination for external validation and coefficient of variation (CV). The prediction accuracy for pH, redox potential, electrical conductivity, moisture, TSS, and ash was good, while for free acidity and HMF it was poor. The results demonstrate that attenuated total reflectance-Fourier transform infrared spectrometry is a valuable, rapid, and nondestructive tool for the quantification of physicochemical properties of honey.

## 1. Introduction

Honey is a natural, sweet, and syrupy fluid collected by bees from the nectar of flowers. The taste and aroma of this liquid vary according to its floral origin and geographical and seasonal conditions. A large number of melliferous sources give therefore the opportunity to produce much characteristically monofloral and a high number of polyfloral nectar honeys [1].

The composition and sensory attributes of honey vary considerably depending on its botanical and geographical origin. Mexico has a great geographical and botanical diversity; for this reason there are many types of blooms that produce different types of honey with a multitude of tastes, colors, and aromas, which are in demand in the European and American markets. Mexico is the fifth largest producer of honey with about 57,000 tons per year and the third largest exporter in the world [2]. The Yucatan Peninsula is the most important honey production region in Mexico; between 30 and 40% of the total amount of Mexican honey for export is obtained from this region. Politically the Yucatan Peninsula consists of the states of Campeche, Quintana Roo, and Yucatan; the state of Campeche produces between 7500 and 4800 ton per year because it has a large botanical biodiversity. The state of Campeche also has two important natural reserves zones. The Reserves Biosphere Petenes is located in the north and the Reserve of Calakmul is located in the southeast of the state. In this botanical diversity that exists in the state of Campeche and the Yucatan peninsula, more than 900 species of flowers producing blooms honey and about 250 species producing pollen have been identified, which contributes to the existence of a great diversity of types of honey in the state of Campeche [3–5].

The physicochemical quality criteria for honey are well specified by* European Council Directive* 110/2001 (EU, 2001) and in the Codex Alimentarius. For the quality control of honey, several physical and chemical properties must be determined, among which we can mention moisture, sugar composition, pH, enzyme activities of invertase and *α*-amylase, hydroxymethylfurfural HMF (mg/kg), electrical conductivity, protein content, insoluble matter, diastase, ash, free acid, total acid, lactonic acid, and redox potential [6, 7]. However, the reference methods used for the quality control of honey are laborious and therefore expensive, thus limiting the number of honey samples analyzed daily. To further improve honey quality control, it is necessary to develop rapid, simple, and accurate methods for routine quality assessment of honey.

In contrast to the time-consuming analysis techniques near infrared spectroscopy (NIRS) and midinfrared spectroscopy (MIR), both vibrational spectroscopy techniques, when combined with multivariate calibration, can be simple, fast execution and low cost [8, 9]. The versatility of Fourier transform infrared (FTIR) spectroscopy and the use of attenuated total reflectance (ATR) simplify the process of spectra acquisition. For these advantages, the Fourier transform infrared (FTIR) spectroscopy and chemometrics methods were used to determine honey adulteration [10–12], while Etzold and Lichtenberg-Kraag [13], Hennessy et al. [14], and Gok et al. [15] employed the infrared spectroscopy and the principal components analysis (PCs), to determine the authentication of botanical geographic origin of honey from Italy, Ireland, Austria, Germany, France, and Turkey. Likewise, the Fourier transform infrared spectroscopic method with attenuated total reflectance (FTIR-ATR) and partial least squares (PLS) regression were used by several types of research for the construction of calibration models to predict physicochemical properties of honey [16–21].

The aim of this study was to determine eight physicochemical properties for Campeche honey, with the aim to develop PLS regression models based on the FTIR-ATR spectroscopy transform for to be used as a rapid and nondestructive analytical tool for quality control of honey.

## 2. Materials and Methods

### 2.1. Honey Samples

**Figure 1:**
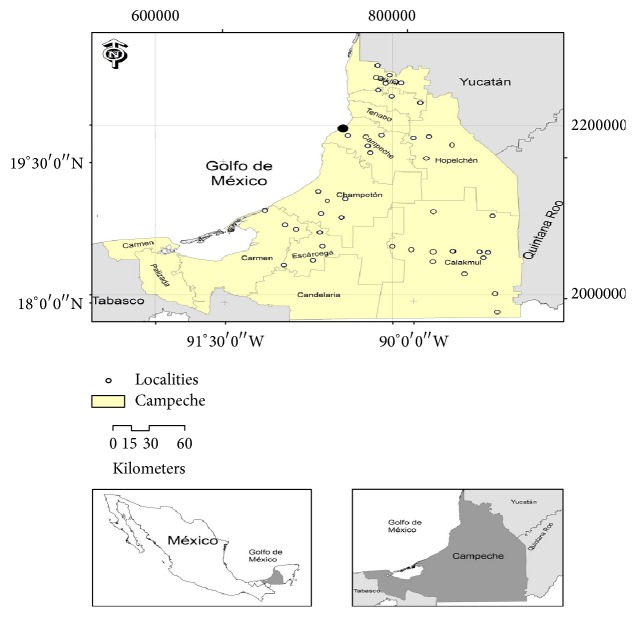
Geographical origin of the honey samples collected from different regions of the state of Campeche, Mexico.

For this work a total of 189 honey samples were collected in 43 communities from eight different municipalities of the state of Campeche, 172 honey samples were of the* Apis mellifera *specie, and 17 samples were of* Melipona beecheii *specie: Calakmul (40), Calkini (27), Campeche Municipality (26), Champoton (20), Escarcega (18), Holpechen (22), Hecelchakan (7), and Carmen (29). These samples were recollected during the major honey production season (Figure 1). The sampling period was conducted between January 2014 to June 2014 and January 2015 to June 2015. All samples were collected from Mayan beekeepers and were transferred to the laboratory at 25°C in dark conditions until analysis. For the honey production season in which the samples honey were collected, the principal blooms were producing nectar that was identified by Mayan beekeepers: Tajonal* (Viguiera dentata)*, Tsitsilche* (Gymnopodium antigonoides)*, Ja'abin* (Piscidia piscipula *L.), Tzalam* (Lysiloma bahamensis)*, Pukté* (Bucida buceras)*, Xa'an, huano* (Sabal yapa)*, Kibix ak'* (Dalbergia glabra (Mill.) Standl)*, Juluup* (Bravaisia berlandieriana)*, Xtabentum* (Turbina corymbosa)*, K'aniste'* (Pouteria campechiana)*, Tinto o Palo Tinto* (Haematoxylum campechianum *L.), Cascarillo* (Cinnamomum porphyrium)*, Chéechem* (Metopium brownei)*, Gusanillo* (Acalypha arvensis Poepp. & Endl)*, Machiche* (Lonchocarpus castilloi)*, Mangle Negro* (Avicennia germinans)*, Bojom* (Cordia alliodora), *Tzuk-tzuc* (Diphysa yucatanensis)*, and Chakaj* (Bursera simaruba)*.

### 2.2. Analysis Honey

The reference methods used for the quantitative determination of free acidity, pH, hydroxymethylfurfural (HMF), electrical conductivity, ash content, moisture content, total soluble solids (TSS), and redox potential of the samples honey were in agreement with the standardized methods proposed by the official methods* (AOAC, International Honey Commission and Codex Alimentarius)* [22–24]; each sample was analyzed in triplicate, for each physicochemical property.

#### 2.2.1. pH Determination

The pH in honey samples was measured in a solution of 10 g of honey in 75 mL ultrapure water free of carbon dioxide, at 20°C using a pH-meter Thermo Scientific, model Orion Star A211. The pH-meter was calibrated using buffer standard solution between 4–7 and 7–10 pH values [22].

#### 2.2.2. Acidity Free

The concentration of free was determined by a titrimetric method [22]. 10 g of honey samples was dissolved in 75 mL of water-free carbon dioxide in a 250 mL beaker. The electrode of pH-meter Thermo Scientific was immersed in the solution, stirred with a magnetic stirrer, and titrated with solution 0.05 N NaOH to pH 8.5 (free acidity). The results were expressed as milliequivalents/kg (meq/kg).

#### 2.2.3. Electrical Conductivity

The electrical conductivity in honey samples was measured at 20°C, by dissolution 20 g honey sample in a 100 mL in ultrapure water with Thermo Scientific conductimeter; the results were expressed as mS/cm [23].

#### 2.2.4. Ash Determination

Ash percent was measured by calcination in muffle furnace Lindberg/Blue, for one night in a furnace at 550°C, until reaching a constant mass [23].

#### 2.2.5. Moisture and Total Soluble Solids (TSS)

The moisture and the total soluble solids in honey samples were determinate based on the refractometry method. The refractometry indexes in honey samples were measured at 25°C using an Atago refractometer model PAL-2SS and the reading was further corrected to a standard temperature of 20°C by adding the correction factor of 0.00023/°C [22, 23]. The moisture was the expressed as weight percent and the TSS in Brix°.

#### 2.2.6. Hydroxymethylfurfural (HMF)

Hydroxymethylfurfural was determined by spectrometry UV-visible, about 5 g honey sample after clarifying with Carrez reagents I and II and the addition of sodium bisulfate [23]. The absorbance was measured at 284 and 336 nm in spectrometer HACH model DR 6000. The concentration of HMF was expressed as mg/kg.

#### 2.2.7. Redox Potential

Redox potential was measured at 20°C using a pH-meter Thermo Scientific, model Orion Star A211. Honey samples were diluted with deionized water, ranging from 10% to 100% (w/v) [25].

### 2.3. Spectroscopic Analysis

The FTIR spectra of honey were acquired with an Agilent Model 660 spectrometer, equipped with a diamond-tip single reflection attenuated total reflectance (ATR), Pike Technologies model Gladi. The software Resolution 4.0 pro Variant served as an interface between the computer and the spectrophotometer. For the analysis of the honey about 0.3 mL of honey was placed on the diamond-tip of ATR; all spectra were recorded at a controlled temperature (24 ± 1°C); triplicate spectra per honey sample were obtained with eight scans per spectrum at a spectral resolution of 2 cm^−1^ in the wavenumber range from 700 to 3700 cm^−1^; after each measurement the ATR crystal surface was cleaned with acetone and dried with absorbent paper. It was necessary to quickly collect the infrared spectrum of the honey samples, because the infrared equipment detects the presence of atmospheric carbon dioxide and increases the measurement error; so it was necessary to perform the analysis of honey using a low number of scans; technically the equipment can operate with eight scans losing quality data collection. The data collected were exported to* Microsoft Excel* 2013 and subsequently exported to the* Infometrix Pirouette V.*4.5 USA, software to build the calibration models using partial least squares (PLS).

### 2.4. Calibration and Validation

**Figure 2:**
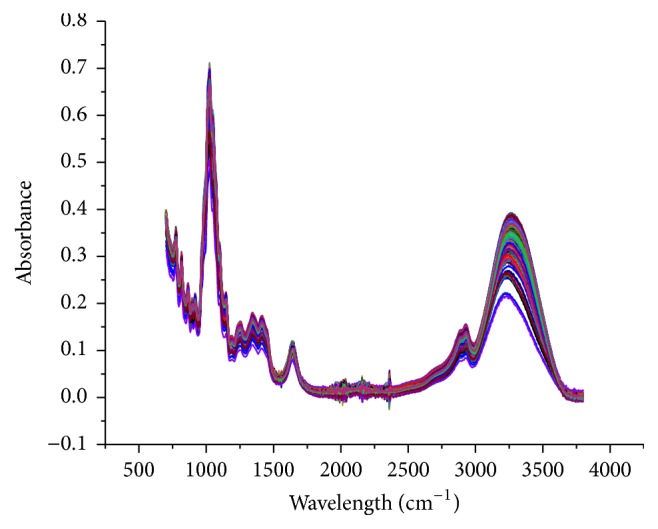
Characteristic of FTIR-ATR spectrum from all honeys samples, acquired from 3700 to 700 cm^−1^.

The Pirouette software was employed for modeling and data treatment. In total 567 spectral fingerprint data obtained from 189 honey samples were used for the construction of each calibration model, in the FTIR region from 700 to 3700 cm^−1^ (Figure 2). Each spectrum consists of 1500 variables (vector, 1–1500), creating a matrix (*X*) of size (567) *∗* (1500) with a total of 850,500 variables. The use of principal components analysis (PCA) permitted the synthesis of information reducing the number of variables to fewer losing the least amount of information possible. Calibration models between the* Y-*variables (reference values) and MIR spectra were developed using PLS analysis with cross-validation and external validation; eight models were built. A total of 151 samples were randomly selected and formed calibration set, and 38 samples were formed to external validation set. Each model was expressed as

(1) $$\begin{array}{c} Y=\beta_{0}+\beta_{1}X_{\lambda 1}+\beta_{2}X_{\lambda 2}+\beta_{3}X_{\lambda 3}+\cdots +\beta_{1500}X_{\lambda 1500}, \end{array}$$

where *β* are the coefficient of the calibration curve, where the correlation coefficient greater than zero is positive or negative if it is less than zero, and *X*_*λn*_ are the wavelengths read every 2 cm^−1^. In this work 1500 coefficients were found for each model.

The preprocessing of the spectra set improves the performance of the calibration models and capacity of predictions. Different treatments were applied to matrix MIR spectra: mean-center, autoscale, baseline correct, normalize, smooth, first derivate, align, logarithm, and standard normal variate (SNV). The reference values determined by chemical methods and the spectral data of both calibration and validation sets were analyzed to detect any outliers using a general Mahalanobis distance (*H*). For each model calibration Mahalanobis distance was calculated from principal components analysis (PCA), and Mahalanobis distance was different for each physicochemical property of honey. Pirouette software helps to determine the Mahalanobis distance to detect outlier points.

To determine the robustness of the calibration models, these models were evaluated using cross-validation with “leave fifteen out” data. The predictive abilities of the models were evaluated through external validation, using a set of 38 samples, and not involved in the construction of the calibration model. To determine the predictive capacity of the calibration models the following statistical parameters were calculated: (PCs) optimum number of principal components, (SECV) standard error of cross-validation, (*R* ^2^_cal_) coefficient of determination of cross-validation, which determines the degree of correlation between the reference values and that predicted by the calibration model, and (*R* ^2^_val_) coefficient of determination for external validation, which determines the correlation between the reference values and that predicted by the validation model and standard error of prediction (SEP); these statistical parameters were also used to choose the optimal calibration equation.

For a better analysis of the standard deviation between the data obtained by the reference methods and predicted by the models calibration of each physicochemical property was necessary to determine the coefficient of variation (CV). The CV is a measure of reproducibility of the model and as a general rule a model can be considered reasonably reproducible if its CV is not greater than 10%.

## 3. Results and Discussion

### 3.1. FTIR Spectral Information

The 189 samples of pure honey were analyzed by triplicate using spectroscopy FTIR-ATR. Figure 2 shows MIR spectrum between 3700 and 700 cm^−1^ of samples honey. The absorption bands between 3700 and 3000 cm^−1^ are due to stretching vibrations of the functional group -OH from carbohydrates, water, and organic acids presents in the honey. The absorption band at 3000–2700 cm^−1^ corresponds to the stretching vibration of bonds C-H that constitutes the chemical skeleton of sugars. The band 1700–1600 cm^−1^ are due to the bending vibrations of -OH from water and stretching vibrations of functional groups ketone C=O of fructose and aldehyde CH=O of glucose. Fingerprint region presents multiple absorbance bands; the bands between 1470 and 700 cm^−1^ are due to the stretching vibrations of bonds C-O, C-C, and C-H and the bending vibrations of C-H present in the chemical structure of carbohydrates [15, 19, 20]. They may also be related to the presence of organic acids, carotenes, and polyphenols [26].

### 3.2. MIR Calibration Models

To construct, the PLS calibration model was used, the FTIR spectral information contained between 3700 and 700 cm^−1^, interference free region. Several pretreatments were realized to spectral fingerprints to reduce error; the Mahalanobis distance was established for each calibration model in order to determine the outliers points Table 1. The results of the cross-validation, external validation, and coefficient of variation are given in Table 2. The prediction of the individual measurements is discussed below.

The results of analysis physicochemical of honey samples for each municipality of the state of Campeche, medium, maximum, and minimum values for each property, are presented in Table 3.

#### 3.2.1. pH and Free Acidity Calibration Models

The pH is a parameter that is correlated with honey storage and with microorganism growth that could change the texture and the honey stability; the pH value can be used for the discrimination of floral and honeydew honey [25]. The pH values of all honey samples ranged from 3.80 ± 0.03 to 4.40 ± 0.03 (Table 3) and were within the limit (pH = 3.4 to 6.1) as described by Moniruzzaman et al. (2013) [27]. Honey samples that were collected in the municipalities of Champoton, Escarcega, Campeche, and Carmen had the lowest pH values in the range 3.80 ± 0.03 to 3.97 ± 0.02. The honeys that were collected in these municipalities come most from the following blooms: Tajonal* (Viguiera dentata)*, Ja'abin* (Piscidia piscipula *L.), Pukté* (Bucida buceras)*, Juluup* (Bravaisia berlandieriana)*, Xtabentum* (Turbina corymbosa)*, K'aniste'* (Pouteria campechiana)*, Tinto o Palo Tinto* (Haematoxylum campechianum *L.), Cascarillo* (Cinnamomum porphyrium)*, Mangle Negro* (Avicennia germinans)*, Bojom* (Cordia alliodora)* and, Tzuk-tzuc* (Diphysa yucatanensis)*. While honey samples of the municipality of Holpechen showed the highest values 4.40 ± 0.03, blooms that are predominantly found in this municipality are Tajonal* (Viguiera dentata)*, Tsitsilche* (Gymnopodium antigonoides)*, Ja'abin* (Piscidia piscipula *L.), Tzalam* (Lysiloma bahamensis)*, Xa'an, huano* (Sabal yapa)*, and Chakaj* (Bursera simaruba)*.

**Figure 3:**
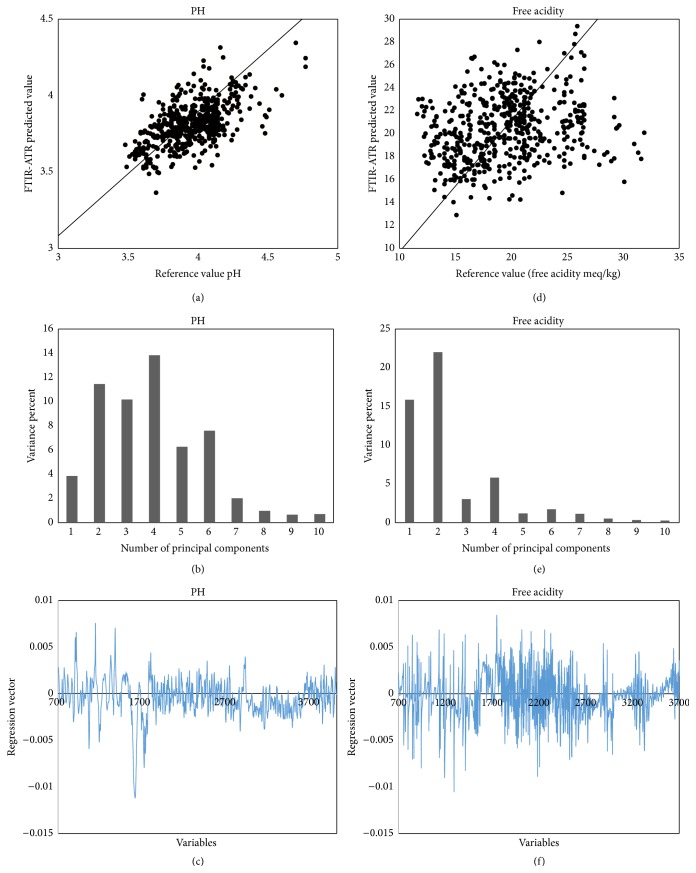
PLS validation plots of pH, contribution of PCA, and regression vector (a)–(c); PLS validation plots of free acidity, contribution of PCA, and regression vector (d)–(f).

Figure 3(a) shows the PLS calibration model for predicting pH in Campeche honey (Table 2). The calibration model presented high values for determination coefficient calibration *R* ^2^_cal_ = 0.904 and prediction *R* ^2^_val_ = 0.842, respectively; low values for standard error calibration cross-validation SECV = 0.093 and standard error prediction SEP = 0.211 and coefficient of variation CV = 5.783% (CV < 10%) show that the calibration model based on infrared spectroscopy successfully predicts the pH to the honey of the state of Campeche. These results partly confirm those obtained by Ruoff et al. (2006) (SECV: 0.12, *R* ^2^_cal_: 0.928, SEP: 0.16, *R* ^2^_val_: 0.868) [18]. The contribution of the principal components is shown in Figure 3(b); as can be seen, the first to sixth principal component had a greater contribution: the second principal component contributes 11.45%, the third principal component contributes 10.17%, and the fourth principal component contributes 13.84%, respectively, and explains 35.46% of the total variance. The variance explained was 57.55% and 42.45% of the unexplained variance could be attributed to the variations in pH presenting the honey samples, experimental errors in the determination of pH using conventional methods, and the collection of spectra. Figure 3(c) shows that the regression vector can observe the contributions the MIR spectral regions that explain the pH in Campeche honey. Four regions can be observed: the first region that is approximately between 3700 and 3000 cm^−1^ is attributed to stretching vibration of functional groups -OH present in sugars, organic acids, and water; the second region between 3000 and 2700 cm^−1^ corresponds to stretching vibration of bonds CH-CH of carbohydrates; the third band between 1700–1500 cm^−1^ is attributable to vibration of bending -OH groups; and the fourth band between 1400 and 900 cm^−1^ shows a diversity of bands attributed to stretching vibrations of bonds C-O, C-C, and C-H and bending vibrations of C-H present in the chemical structure of carbohydrates and organic acids [15, 19, 20].

Honey acidity is due to the presence of organic acid, mainly gluconic acid, and to inorganic ions such as sulfate, phosphate, and chloride. Acid measurement is useful for evaluation of honey fermentation, authentication of unifloral honey, and differentiating nectar from honeydew [28]. The results of the analysis of honey samples of honey collected in the state of Campeche had values of free acidity 15.77 ± 0.74 to 23.03 ± 0.54 meq/kg (Table 3). Neither sample showed values greater than 50 meq/kg* Codex Alimentarius* 2001 [24], indicating that no sample of honey presented fermentation and is fresh. Figure 3(d) shows the calibration model for free acidity and Table 2 shows the statistical parameters (SEP: 4.203 meq/kg, *R* ^2^_val_: 0.668); moreover, the coefficient of variation to free acidity was CV = 21.061% (CV > 10%); this statistical values show that the ability of the model prediction for free acidity has a poor accuracy. This is attributed to the fact that at very low concentrations of a chemical species the infrared absorbance bands are very small, which makes the determination of organic acids with a good precision difficult. While honey contains high concentrations of sugar and water, their functional groups are detected with greater intensity in the spectrum MIR. Our results of free acidity are in agreement with the results obtained by Qiu et al. (1999) (SEP: 4.39 meq/kg, *R* ^2^_val_: 0.49) of bee honey produced in eleven countries [29]. Figure 3(e) shows the contribution of the principal components for calibration model to free acidity. The first and second principal components explain 39% of the variance of the data, while 10 principal components explain only 51.96% of the variance. The regression vector (Figure 3(f)) shows three regions used predict to quantify organic acids. The first spectral region between 3700 and 3000 cm^−1^ corresponds to the vibrational mode stretching -OH functional group present in the organic acids; the second spectral band between 1700 and 1500 cm^−1^ is related to the stretching vibration of the C=O functional group present in the acetic and gluconic acids and originating from the fermentation of sugar. The third region between 1400 and 900 cm^−1^ shows several bands absorbance related to vibration bending of C-C-H, C-O-H, and O-C-H and stretching vibrations of C-O and C-C, which constitute the chemical structure of the sugar and principally glucose and fructose.

#### 3.2.2. Electrical Conductivity and Ash Calibration Models

**Figure 4:**
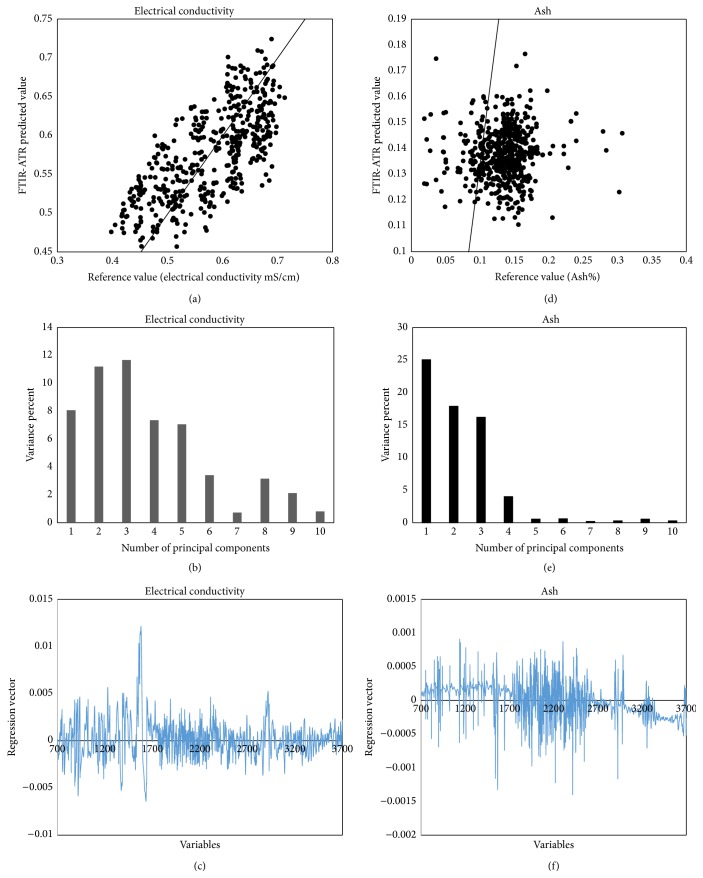
PLS validation plots of electrical conductivity, contribution of PCA, and regression vector (a)–(c); PLS validation plots of Ash, contribution of PCA, and regression vector (d)–(f).

The electrical conductivity depends on the ash, organic acid, mineral compounds such as sulfates and phosphates, protein, and some complex sugar and polyols contents and varies with botanical origin. Electrical conductivity is widely used for discrimination between honeydew and blossom and also for the characterization of unifloral honey [30]. The electrical conductivity is correlated with the mineral composition of the honey so that electrical conductivity can be used as a criterion to determine the botanical origin of honey [31, 32]. Honey samples of Hecelchakan and Calkini municipalities presented the greatest values of electrical conductivity (0.61 ± 0.02 and 0.68 ± 0.01) mS/cm and ash (0.15 ± 0.20 and 0.16 ± 0.11%), respectively (Table 3). This is attributed because honeys collected in these municipalities are meliponas honey (*Melipona beecheii*), while the samples of* Apis mellifera* honey showed values of electrical conductivity of 0.59 ± 0.01 to 0.48 ± 0.05 mS/cm and ash content of 0.15 ± 0.09 to 0.12 ± 0.08% (Table 3). The values of electrical conductivity had lower values than 0.8 mS/cm, which is the maximum allowable value, while the values of the percentage of ash in all honey samples analyzed were less than 0.6% limit in the standard* Codex Alimentarius *(2001) [24].  Figure 4(a) shows the calibration model for electrical conductivity. Table 2 shows high values for the determination coefficient of cross-validation *R* ^2^_cal_ = 0.968 and determination coefficient for external validation *R* ^2^_val_ = 0.862. On the other hand, small values of the standard error of cross-validation (SECV = 0.021 mS/cm) and of the standard error prediction of external validation (SEP = 0.109 mS/cm) and a value of the coefficient of variation (CV = 8.583%) 8 which is less than 10 indicate that the calibration model has good capacity to predict the electrical conductivity in Campeche honey. The results obtained in this work are in concordance with reported data by Ruoff et al. (2006) (SEP: 0.01 mS/cm, *R* ^2^_val_: 0.88; SEP: 0.14 mS/cm, *R* ^2^_val_: 0.87) and Cozzolino and Corbella (2003) (SEP: 0.01 mS/cm, *R* ^2^_val_: 0.88) and [18, 31]. The contribution of the principal components of the calibration model for predicting electrical conductivity in honey Figure 4(b) shows that 1 to 6 were the principal components and was the greatest contribution model calibration and can explain the 45.33% of the total variability. The cumulative variance with the ten principal components the model explains 55.54% of the variance.

Figure 4(d) shows the PLS model for content ash. High values for determination coefficients *R* ^2^_cal_ = 0.984 and *R* ^2^_val_ = 0.861 and low values to SECV = 0.008 and SEP = 0.041% (0.041 g/100 g) and coefficient of variation CV = 7.849% (CV < 10%) (Table 2) show that the calibration model to predictive ash content in Campeche honey had good predictive capacity. Our results are similar to those reported by Cozolino and Corbella (2003) for Uruguayan honey (SECV: 0.07, *R* ^2^_cal_: 0.83; SEP: 0.08, *R* ^2^_val_: 0.80 and SECV: 0.04, *R* ^2^_cal_: 0.95; SEP: 0.05, *R* ^2^_val_: 0.90, resp.) [31].  Figure 4(e) shows the contribution that had the principal components for the calibration model for predicting the ash content; the first four components can explain the greatest variance of data 63.17% and with 10 principal components can explain 65.68% of the total variance in predicting ash content. Unexplained variance to electrical conductivity and ash content could be attributed to the great botanical diversity that exists in the state of Campeche and experimental errors.

Figures 4(c) and 4(f) show the regression vectors, for calibration models for prediction of electrical conductivity and ash content. The regression vectors for electrical conductivity and ash have three spectral regions of importance in the MIR showing chemical information useful in the construction of calibration models: the first region located between 3700 and 3200 cm^−1^ is attributed to stretching vibration of -OH and the second region between 1700 and 1500 cm^−1^ had a great contribution; the bands are attributed to bending -OH vibrations present in the water and are a component present in high concentration in honey. The third region between 1400 and 900 cm^−1^ shows that it may be related to stretching vibrations and bending functional groups C-C, C-O, O-C-H, and C-O-H constituting the chemical skeleton of the sugar, polyphenols, and organic acids [25].

#### 3.2.3. Total Soluble Solids (TSS) and Moisture

**Figure 5:**
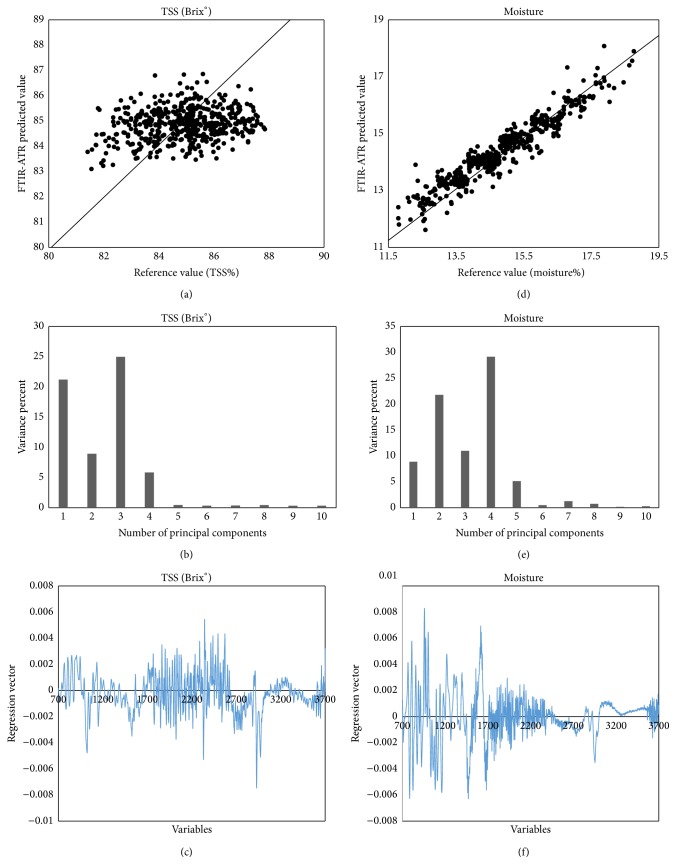
PLS validation plots of TSS (Brix°), contribution of PCA, and regression vector (a)–(c); PLS validation plots of moisture contribution of PCA and regression vector (d)–(f).

The Brix° scale is used in the food industry for measuring the approximate amount of sugars. The honey is mainly composed of sugars; about 25 different oligosaccharides have been detected in the composition of honey. The fructose and glucose are present in a higher concentration and provides the honey with its extreme sweetness. The total soluble solids (TSS) which are directly related to sugar content may be a reliable index of adulteration. Honey samples municipalities like Calkini and Hecelchakan had the lowest concentration of sugar, 82.44 ± 0.46 and 81.06 ± 0.25%, respectively (Table 3). The low concentration of sugar in these samples is because they are meliponas honey (*Melipona beecheii*), and as already explained above they are honey containing high concentrations of moisture, while the samples of honey of the species* Apis mellifera *presented concentrations of sugar between 84.18 ± 0.21 and 86.06 ± 0.25%. The (TSS) results obtained in this study indicate that the honey of the state of Campeche has no adulteration with any kind of commercial sugar. The results of the calibration model for the prediction of TSS (Figure 5(a) and Table 2) show high value of coefficient of determination *R* ^2^_cal_ = 0.965 and *R* ^2^_val_ = 0.859 and low standard errors for both cross-validation SECV = 0.38% and prediction SEP = 1.84% and low value to coefficient of variation CV = 5.314% (CV < 10%), indicating that the model based on infrared spectroscopy has good accuracy to determine TSS. These data are consistent with those the reported by Ruoff et al. (2006) who reported values for *R* ^2^_val_ (0.81 and 0.884) for prediction of glucose and fructose, respectively [18]. Figure 5(b) shows that 1–4 principal components are the most weight in building the calibration model to predict the content of TSS and can explain 60.96% of variance. Also the total variance explained by the model was 63.24%. Figure 5(c) shows the vector regression model that predicts the total sugar content. The useful chemical information that was used to build the calibration model for predicting total sugars can be attributed to four regions: the first region located between 3700 and 3200 cm^−1^ is related to stretching vibrations of -OH functional groups constituting the molecules of sugars, organic acids, and water; the second region located between 3000 and 2700 cm^−1^ is related to vibration stretching of bonds C-H; the third region located between 1700 and 1500 cm^−1^ corresponds to stretching vibrations of functional group aldehyde CH=O and the functional group ketones C=O, present in the molecules of glucose and fructose, respectively; the fourth region presented multiple bands between 1400 and 750 cm^−1^ stretching vibration of bonds C-H, C-O, and C-C and vibration bending of C-H bonds which constitute the chemical skeleton of sugars.

The honey moisture content depends on the environmental conditions, and also the honey degree of maturity and the manipulation from beekeepers at the harvest period, and it can vary from season to season and from year to year. The moisture is an important parameter of control quality in honey; higher moisture content could lead to undesirable honey fermentation during storage. The honey fermentation produces the formation of ethyl alcohol and carbon dioxide; the alcohol can be oxidized to form acetic acid and water, which is undesirable because it alters the quality of the honey [25]. The moisture content in Campeche honey varied in the range between 14.15 ± 0.08 and 18.94 ± 0.25% (Table 3). Honey samples that recorded higher moisture content were collected in the municipalities of Hecelchakan 17.56 ± 0.46 and 18.94 ± 0.25% Calkini, respectively. These honeys had higher moisture because they are meliponas honey* (Melipona beecheii)*; by its nature this type of honey has high concentrations of moisture. Honey samples of* Apis mellifera* had a moisture content that ranged from 14.15 ± 0.18 to 15.82 ± 0.21%. However, all samples collected from the state of Campeche had moisture concentrations below 20%, which is the maximum prescribed limit for the moisture content in honey* Codex Alimentarius* (2001) [24]. The moisture content in Campeche honey is related to weather conditions, maturity, and type of honey. The moisture content of honey varies from season to season and from year to year. The prediction results for the calibration model to determine the moisture are shown in Figure 5(d) and Table 2; high value for the determination coefficients of calibration *R* ^2^_cal_ = 0.979 and validation *R* ^2^_val_ = 0.971 and low values for the standard error prediction SEP = 1.2 (1.2 g/100 g) and coefficient of variation CV = 6.343% (CV < 10%) indicate good prediction capacity of moisture in honey samples studied in this work. Similar results were obtained in previous work by Ruoff et al. (2006) for Switzerland honey [18]. To construct the calibration model for predicting the moisture content in the honey, Figure 5(e) shows five major components that had the largest contribution and help explain 75.9% of the variance of the data. The total variance explained by the model calibration was 78.88%. The regression vector (Figure 5(f)) shows four absorbance bands. The first band between 3700 and 3000 cm^−1^ is related to stretching vibrations and the third band between 1700 and 1500 cm^−1^ corresponds to bending vibrations of the -OH group, respectively, constituting the water molecules, organic acids, and sugars. The second region between 3000 and 2700 cm^−1^ and the fourth region 1400–750 cm^−1^ corresponding to multiple absorbance bands of functional groups are molecules of sugars and organic acids.

#### 3.2.4. HMF and Redox Potential Calibration Models

**Figure 6:**
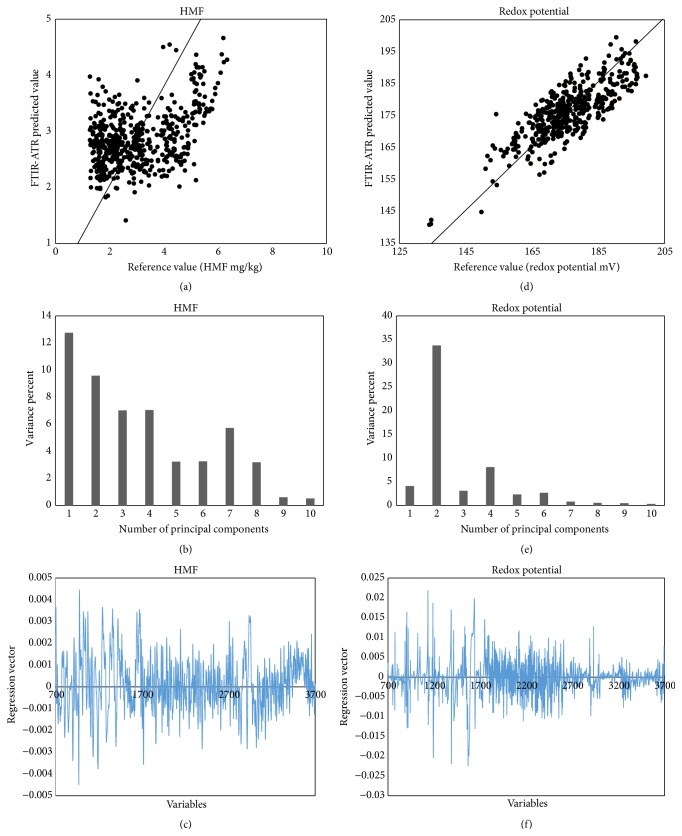
PLS validation plots of HMF, contribution of PCA, and regression vector (a)–(c); PLS validation plots of Redox potential, contribution of PCA, and regression vector (d)–(f).

The HMF content is widely recognized as an indicator of freshness and related to storage, processing, and possible adulteration with invert syrup, unrelated to the botanical or geography origin. Several factors influence the levels of HMF, such as temperature, time of heating, pH, storage conditions, and floral source [6]. All honey samples collected in different municipalities of the state of Campeche showed low levels of HMF in a range between 2.45 ± 0.11 and 4.45 ± 0.53 mg/kg (Table 3); these values are below the allowed limit which is 40 mg/kg. The results show that the honey of the state of Campeche are fresh honey, which was to be expected as they are honey that is less than 6 months after being collected. The PLS model was developed to predict the HMF which presented high (*R* ^2^_cal_ = 0.982 and *R* ^2^_val_ = 0.961); however high value to SEP = 29.18 mg/kg and the coefficient of variation CV = 43.217% (CV > 10%) (Table 2) show that the model for HMF had poor accuracy to predict the HMF in honey. This is attributed to the fact that honey contains a low concentration of HMF in honey making it difficult to detect by infrared spectroscopy. Principal components 1 to 8 were those that had the greatest contribution in building the calibration model to predict the concentration of HMF in honey (Figure 6(b)). The variance explained by the model was a low 52.92%; this is attributed to low concentrations of HMF; the infrared spectroscopy is not efficient for the quantification of HMF and experimental errors in the quantification of HMF by conventional methods. Figure 6(c) shows the vector regression calibration model that predicts the HMF concentration. The HMF molecule consists of a ring having unsaturated bonds and furan functional group and two branches, one containing an aldehyde functional group and the other containing the functional group -OH. The vector regression has several absorbance bands that could be attributed to the absorbance of these functional groups [26].

The redox potential gives information about the real oxidation/reduction ability of a molecule and its prevalent form oxidized or reduced in the system. Therefore, the redox potential may be also an interesting indicator of the antioxidant efficiency of food [25, 33]. Oxidation-reduction reactions in honey which take place in unsaturated bonds of the molecules of sugars, glucose, and fructose have unsaturated bonds in their aldehyde functional groups CH=O and ketone C=O; these atoms can capture or transfer one or more electrons to the environment causing physical and chemical changes in the honey. Thus the redox potential can help identify these changes in the food during storage. Likewise, the redox potential may be an indicator of the content of antioxidants in honey. Redox potential in Campeche honey was between 151.03 and 187.96 ± 1.62 ± 1.70 mV (Table 3). Honey that was collected in the municipalities of Holpechen, Hecelchakan, Calakmul, and Calkini had lower values redox potential, and this could indicate that these honeys are chemically more stable, possibly because they have a higher concentration of phenolic and carotenes; this is attributable to the fact that most of the samples of honey from these municipalities had deep red color to black color. While the honey of the municipalities of Campeche, Escarcega, Carmen, and Champoton had higher values redox potential 177.32 ± 1.49 to 187.96 ± 1.70 mV, this is also due the fact that these honeys have fewer concentrations of antioxidants and carotenes and higher concentrations of organic acids as were the honey of these municipalities which had lower values pH (Table 3). Redox potential can be used to measure changes in the chemical composition of honey due to changes in the chemical structure of sugars. Redox potential can also be used to determine the degree of concentration of antioxidant compounds such as polyphenols and carotenes present in honey. Samples of honey from the Holpechen municipality had the lowest values redox potential which could indicate that they are honey that have greater concentration of antioxidants, while on the opposite, samples of honey from the municipalities of Champoton, Carmen, Escarcega, and Campeche had the highest values in the redox potential. The graphical representation of the calibration model for the redox potential is shown in Figure 6(d). The coefficients of determination of calibration and external validation presented satisfactory values (*R* ^2^_cal_ = 0.98 and *R* ^2^_val_ = 0.816, resp.), whereas small values were found for the standard error cross-validation (SECV = 1.473 mV) and standard error of prediction (SEP = 1.337 mV) and coefficient of variation (CV = 5.593%), indicating that the redox potential in honey can be predicted by infrared spectroscopy with a satisfying accuracy. The calibration model for predicting the redox potential in honey (Figure 6(e)) shows that the second principal component was the largest contribution and can explain the 33.77% of the variance of this property, and the model explained 55.54% of the total variance. The Figure 6(f) shows the spectral information used to construct the calibration model to predict the redox potential. The regions 3700–3000 cm^−1^, 3000–2700 cm^−1^, and 1400–800 cm^−1^ correspond to stretching and bending vibration that could be associated with the presence of the functional groups -OH, aldehydes CH=O, C=O ketones, and bonds O-C-O, C-H, C-O, and C-H, constituting the molecules of sugar, polyphenols, and carotenes [26].

## 4. Conclusion

The ATR-midinfrared spectroscopy combined with chemometrics techniques based on PLS makes the development of calibrations models that have a satisfactory power prediction for the quality parameters of honey studies possible: pH, electrical conductivity, ash, moisture, TSS (Brix°), and redox potential. The honeys of the state of Campeche presented low concentrations of organic acids and HMF, so that the absorbance bands of their functional groups in the infrared spectrum were not useful, to be able to construct calibration models that allowed predicting insatisfactorily the free acidity and HMF in the honeys of the state of Campeche.

The determination of measurands such as pH and redox potential is valuable for the evaluation of storage and heat damage, while the determination of measurands for electrical conductivity and ash could allow determining the geographical origin of honey. Redox potential could be used to determine the concentration of antioxidants in Campeche honeys.

The main advantage of ATR-MIR combined with chemometrics methods is that it can provide valuable information of several measurands used for quality control of honey from the state of Campeche, with the advantage that is fast, low cost, and environment friendly.

## Figures and Tables

**Table 1:** PLS calibration models were developed applying different mathematics treatments to reduced errors in predicting of the properties of honeys.

Honey properties	Different spectral treatments sequence	Mahalanobis distance, criterion	Outliers points
pH	Autoscale, baseline correct (quadratic), normalize, smooth (order polynomial 25)	*H* ≥ 7.9	23
Free acidity	Autoscale, 1st derivate (order polynomial 15), log_10_, baseline correct (quadratic), normalize	*H* ≥ 7.9	43
Electrical conductivity	Autoscale, baseline correct (quadratic), normalize, 1st derivate (order polynomial 15), smooth (order polynomial 25)	*H* ≥ 12.08	27
Ash	Autoscale, 1st derivate (order polynomial 15), log_10_, normalize, baseline correct (quadratic)	*H* ≥ 7.9	23
TSS (Brix°)	Mean-center, log_10_, baseline correct (quadratic), normalize, smooth (order polynomial 25)	*H* ≥ 7.9	21
Moisture	Autoscale, baseline correct (quadratic), log_10_, normalize, align (15)	*H* ≥ 12.9	19
HMF	Autoscale, baseline correct (quadratic), log_10_, 1st derivate (order polynomial 15), align (15)	*H* ≥ 6.04	22
Redox potential	Autoscale, 1st derivate (order polynomial 15), log_10_, baseline correct (quadratic), normalize	*H* ≥ 12.08	19

**Table 2:** Statistical parameters obtained for each of the calibration models.

Honey properties	PCs	SECV	*R* ^2^_cal_	PCs	SEP	*R* ^2^_val_	Coefficient of variation
pH	4	0.093	0.904	6	0.211	0.842	5.783
Free acidity	2	1.257	0.973	3	4.203	0.668	21.061
Electrical conductivity	2	0.021	0.968	2	0.109	0.862	8.583
Ash	2	0.008	0.984	1	0.041	0.861	7.849
TSS (Brix°)	2	0.380	0.965	4	1.846	0.859	5.314
Moisture	5	0.298	0.979	5	1.205	0.971	6.343
HMF	3	0.474	0.982	3	29.18	0.961	43.217
Redox potential	2	1.473	0.984	4	1.337	0.816	5.593

**Table 3:** Physicochemical for honey samples of Campeche state.

Municipalities	pH	Free acidity (meq/kg)	Electrical conductivity (mS/cm)	Ash (%)	TSS (Brix°)	Moisture (%)	HMF (mg/kg)	Redox potential (mV)
Calakmul	4.03 ± 0.02	16.2 ± 0.44	0.58 ± 0.03	0.14 ± 0.06	84.86 ± 0.15	15.14 ± 0.15	3.28 ± 0.56	173.26 ± 1.20
Calkiní	4.09 ± 0.01	15.77 ± 0.74	0.68 ± 0.01	0.16 ± 0.11	86.06 ± 0.25	18.94 ± 0.25	2.73 ± 0.23	173.53 ± 2.04
Campeche	3.95 ± 0.04	23.03 ± 0.54	0.48 ± 0.05	0.12 ± 0.08	85.85 ± 0.18	14.15 ± 0.18	3.34 ± 0.64	177.32 ± 1.49
Champotón	3.80 ± 0.03	22.81 ± 0.62	0.55 ± 0.07	0.13 ± 0.09	84.18 ± 0.21	15.82 ± 0.21	2.86 ± 0.46	187.96 ± 1.70
Escarcega	3.88 ± 0.05	22.72 ± 0.67	0.58 ± 0.01	0.14 ± 0.10	84.97 ± 0.22	15.03 ± 0.22	2.45 ± 0.11	181.43 ± 1.80
Hopelchén	4.40 ± 0.03	15.82 ± 0.59	0.59 ± 0.01	0.15 ± 0.09	85.28 ± 0.20	14.72 ± 0.20	3.15 ± 0.19	151.03 ± 1.62
Hecelchakán	4.01 ± 0.06	16.23 ± 0.38	0.61 ± 0.02	0.15 ± 0.20	82.44 ± 0.46	17.56 ± 0.46	4.45 ± 0.53	170.49 ± 3.81
Sabancuy	3.97 ± 0.02	21.55 ± 0.51	0.57 ± 0.04	0.13 ± 0.08	84.98 ± 0.17	15.02 ± 0.17	3.91 ± 0.35	186.03 ± 1.42
